# Coagulopathy in Acute Puumala Hantavirus Infection

**DOI:** 10.3390/v13081553

**Published:** 2021-08-06

**Authors:** Sirpa Koskela, Satu Mäkelä, Tomas Strandin, Antti Vaheri, Tuula Outinen, Lotta Joutsi-Korhonen, Ilkka Pörsti, Jukka Mustonen, Outi Laine

**Affiliations:** 1 Department of Internal Medicine, Tampere University Hospital, Elämänaukio 2, 33520 Tampere, Finland; satu.makela@pshp.fi (S.M.); tuula.outinen@pshp.fi (T.O.); ilkka.porsti@tuni.fi (I.P.); jukka.mustonen@tuni.fi (J.M.); outi.laine@pshp.fi (O.L.); 2 Department of Virology, Medicum, University of Helsinki, Haartmaninkatu 21, 00014 Helsinki, Finland; tomas.strandin@helsinki.fi (T.S.); antti.vaheri@helsinki.fi (A.V.); 3 Department of Clinical Chemistry, University of Helsinki and Helsinki University Hospital, 00290 Helsinki, Finland; lotta.joutsi-korhonen@hus.fi; 4 Faculty of Medicine and Health Technology, Tampere University, 33014 Tampere, Finland

**Keywords:** Puumala hantavirus, coagulation, complement system, fibrinolysis, endothelium, platelet, acute kidney injury, DIC

## Abstract

Puumala hantavirus (PUUV) causes a hemorrhagic fever with renal syndrome (HFRS), also called nephropathia epidemica (NE), which is mainly endemic in Europe and Russia. The clinical features include a low platelet count, altered coagulation, endothelial activation, and acute kidney injury (AKI). Multiple connections between coagulation pathways and inflammatory mediators, as well as complement and kallikrein–kinin systems, have been reported. The bleeding symptoms are usually mild. PUUV-infected patients also have an increased risk for disseminated intravascular coagulation (DIC) and thrombosis.

## 1. Introduction

Hantaviruses can cause HFRS in Europe and Asia and hantavirus cardiopulmonary syndrome (HCPS) in America [1,2]. PUUV, carried by the bank vole (*Myodes glareolus*), causes a mild form of HFRS mainly in Northern Europe and Russia [3]. Finland has the highest globally documented incidence of a diagnosed hantavirus disease: in a population of 5.5 million, 1000–3000 cases of HFRS occur annually [4]. Over 10,000 individuals are diagnosed with HFRS every year in Europe [1]. PUUV infection, also called nephropathia epidemica (NE), is typically associated with thrombocytopenia, increased capillary permeability causing vascular leakage, and acute kidney injury (AKI). The reported case fatality rates are up to 10% for Dobrava virus-caused HFRS and as high as 40% for HCPS [1,5]. The mortality of NE is low and has been reported to vary from 0.1% in Finland to up to 0.4% in Sweden [6,7].

Both hemorrhage and thrombosis have been associated with acute PUUV infection [8,9]. The disease pathogenesis is complex and not fully understood, but it is considered to be immune-mediated, with increased levels of inflammatory mediators [4,10]. Along with thrombocytopenia, the hematological abnormalities involve coagulation abnormalities and increased fibrinolysis and complement activation [11,12,13,14,15]. Hantaviruses infect and replicate in the endothelial cells of human capillaries, which ultimately disrupts the balance of regulated hemostasis [4,16,17,18]. Activated platelets and endothelial dysfunction are thus involved in the disease pathogenesis [11,18,19,20,21,22].

In this review, we summarize the current knowledge about the alterations in the coagulation and complement systems that are associated with thrombocytopenia and clinical disease during acute PUUV infection.

## 2. Puumala Hantavirus Infection

### 2.1. Clinical Features of the Disease

The clinical HFRS disease varies from a subclinical to severe outcome. Usually, the incubation period of NE is 2–6 weeks [1]. The clinical course can be divided into febrile, hypotensive, oliguric, polyuric, and convalescent phases, although all phases may not be clinically evident [5,6]. The initial symptoms of the disease include an abrupt high fever and headache, followed by gastrointestinal symptoms, nausea, vomiting, abdominal pain, and backache [1,2]. AKI is one of the hallmarks in NE [6,23]. Ocular and central nervous system symptoms are common in the early phase of the infection [24,25]. Complete recovery after NE is common; however, long-term consequences may include renal impairment and elevated blood pressure [26]. In addition, hormonal deficiencies, such as hypopituitarism, primary hypothyroidism, and hypogonadism, have been described after NE [27].

### 2.2. Acute Kidney Injury

Renal involvement in acute PUUV-HFRS, including transient proteinuria, microscopic hematuria, and AKI, usually begins on the third or fourth day of the illness. During the second week, oliguria is followed by polyuria and usually a full recovery of the renal function. Hemodialysis treatment is needed in 4–6% of the hospitalized patients [23]. AKI, as evaluated by the elevated serum creatinine level, is found in 84% of hospital-treated patients [23].

The typical renal histological finding is acute tubulointerstitial nephritis. Hantaviruses can infect tubular epithelial cells, glomerular endothelial cells, and podocytes of the human kidney, and disrupt cell-to-cell contacts in these cells. This is supposed to diminish the barrier function of the kidney, and thus be the cause of proteinuria [28]. The lack of histological endothelial cell damage suggests that the loss of barrier function is due to inflammation or cytokines instead of endothelial cell death [5,29]. A high degree of proteinuria is a special feature of PUUV-associated tubulointerstitial nephritis [23]. A distinct feature is also medullary hemorrhages, which have been found in 20–60% of acute phase renal biopsies [23]. The presence of hemorrhages in a renal biopsy specimen is a quite specific finding and should alert the pathologist to the possibility of a hantavirus infection.

Several markers indicating the severity of AKI have recently been described [23]. It is unclear how much these biomarkers reflect the inflammatory response to the virus clearance or tissue damage. Interleukin-6 (IL-6), a cytokine produced by various cells, is elevated during acute NE and is associated with severe AKI [30]. Increased pentraxin-3 (PTX-3), a rapid marker of local activation of innate immunity and inflammation, associates with AKI and a low platelet count [31]. Furthermore, indoleamine 2,3-dioxygenase (IDO), the soluble urokinase-type plasminogen activator receptor (suPAR), and GATA-3, have been reported to associate with severe PUUV-induced AKI [32,33,34].

### 2.3. Capillary Leakage

Increased vascular permeability in various organs is characteristic of the pathogenesis of hantavirus infections [6,16]. Plasma leakage from vasculature into tissues explains many clinical features, such as hemoconcentration, hypotension and shock, abdominal symptoms due to retroperitoneal edema, and pleural effusion [16,29]. Data suggests that the endothelial barrier function is impaired because of an enhanced permeability instead of direct cellular cytotoxicity or the injury of vascular cells. In a Finnish study with 546 patients with PUUV infection, thrombocytopenia was associated with clinical and laboratory variables reflecting severe capillary leakage [11]. Current knowledge implies that several simultaneous factors are involved in the increased vascular permeability [35]. The extent of the capillary leakage in the hantavirus infection is influenced by virus characteristics, viral load, and host factors [5].

### 2.4. Hemorrhagic Manifestations

Bleeding symptoms are mild in NE. A decreased platelet count may predispose to bleedings. Petechiae of skin and mucosa, ecchymoses, conjunctival bleedings, and epistaxis are seen in one-third of the NE patients. In addition, conjunctival bleeding, metrorrhagia, macroscopic hematuria, melena, and hematemesis may occur [5,6]. When gastroscopy was performed to ten consecutive patients with acute PUUV infection, a hemorrhagic gastropathy was observed in all of them [36]. In addition, severe and even fatal hemorrhages of the pituitary gland, kidneys, heart, liver, lungs, and peritoneal cavity have been reported [37,38]. A complicated case of spleen hemorrhage has also been described [39].

## 3. Thrombocytopenia in PUUV Infection

### 3.1. Thrombopoiesis and Platelet Activation

A low platelet count seems to be caused by increased platelet destruction in PUUV infection. Bone marrow examinations have shown an increased amount and size of megakaryocytes during acute NE [40]. An increased mean platelet volume and immature platelet fraction, together with an elevated thrombopoietin level, also refer to an active thrombopoiesis in the bone marrow [19,20,41]. An enlarged spleen is a frequently detected lymphoid organ involvement in NE patients. Splenomegaly associates with inflammatory laboratory variables, inversely with high leukocyte count and directly with the CRP level, but not with a low platelet count. Thus, the enhanced splenic sequestration of platelets as a cause for a lowered platelet count does not seem to play a significant role in PUUV infection [42].

The interaction of platelets with PUUV-infected endothelium is suggested to underlie the lowered platelet count [5,17]. The cellular entry of hantaviruses is mediated by β3-integrins on platelets, endothelial cells, and macrophages [43]. Hantavirus interacts with platelets via the GPIIb/IIIa (also known as integrin αIIbβ3) receptor, which contributes to the binding of quiescent platelets to the infected endothelium, platelet activation, viral dissemination, and induction of endothelial responses [17]. Platelet activation contributes to conformation changes in integrin GPIIb/IIIa, enabling it to bind fibrinogen, vWF, and fibronectin that are required for platelet aggregation [44]. These events are suggested to contribute to the decreased amount of circulating platelets and to an increased vascular permeability.

Lowered levels of disintegrin and metalloproteinase with a thrombospondin type 1 domain 13 (ADAMTS13), along with altered platelet ligands of αIIbβ3, such as an elevated von Willebrand factor (vWF), fibrinogen, and decreased fibronectin, have been reported during acute PUUV infection [13]. vWF is produced in the endothelium and megakaryocytes and then carried in the α-granules of the platelets. The primary function of vWF is to mediate platelet adhesion and aggregation to the injured endothelium in both primary hemostasis and thrombosis [45]. An elevated level of circulating vWF may imply endothelial injury and platelet adhesion to vasculature, but also the release of vWF from activated platelets in the acute phase [13]. Fibrinogen, an acute phase protein and a platelet ligand of αIIbβ3, contributes to fibrin clot formation due to thrombin in vascular and tissue injury. Increased fibrinogen may reflect an acute phase reaction in the liver that is strong enough to outweigh the consumption because of ongoing coagulation activity during the acute phase of NE [12,13]. Increased fibrinogen correlates to the decreased platelet count, which further implicates platelet activation and consumption. Enhanced platelet adhesion via vWF and altered platelet ligands thus imply increased platelet adhesion and activation during NE, which may result in platelet consumption and the encountered thrombocytopenia [13]. In summary, the mechanisms of thrombocytopenia remain elusive, but we expect that altered platelet–endothelial cells interactions are involved. The severity of thrombocytopenia is not associated with the upcoming AKI. Interestingly, glucosuria, which sometimes appears early during acute PUUV infection, predicts thrombocytopenia in NE [46].

Elevated levels of platelet receptors, soluble P-selectin, and glycoprotein VI, a collagen receptor mediating platelet activation at the site of vascular injury where collagen is exposed, have been shown in acute NE. These in vivo markers for platelet activation were especially increased in Swedish patients with disseminated intravascular coagulation (DIC) and thrombosis [20]. When assessed in the whole blood using impedance aggregometry Multiplate^®^, platelet aggregation in NE was impaired and correlated with the low platelet count. In addition, platelet aggregation was found to be defective among patients with near normal platelet counts. However, platelet adhesion to the collagen surface was intact when studied by a platelet function test, PFA-100^®^ [19].

### 3.2. Decreased Platelet Count Association with Pentraxin-3, Cell-Free DNA, and Interleukin-6 Levels

A low platelet count has been linked to various inflammatory markers reflecting the disease severity in NE. Increased levels of circulating plasma cell-free deoxyribonucleic acid (cf-DNA) have been reported to associate with thrombocytopenia and leukocytosis during acute disease [47]. Cell-free DNA originates from apoptotic and necrotic cells indicating the amount of cellular damage [48]. However, the urinary excretion of cf-DNA is not increased and does not seem to correlate with the severity of AKI. Elevated levels of cf-DNA possibly reflect the apoptosis occurring in the acute phase, as the levels correlate with the cf-DNA band intensity in the quantitative analysis [47]. IL-6 is a proinflammatory cytokine responsible for acute phase reactants and inflammatory responses by activating T cells and promoting B cell differentiation [49]. In NE, increased plasma IL-6 levels associate with both severe AKI and more severe thrombocytopenia [30,50,51]. High plasma suPAR levels are also found to associate with thrombocytopenia [33].

Pentraxin-3 (PTX-3), an acute phase protein, is generated at the site of inflammation in various cells and tissues, mainly by dendritic cells and macrophages in response to inflammatory signals [52]. PTX-3 recognizes different pathogens, bacteria, viruses, and fungi, modulates complement activity by binding C1q, and facilitates pathogen recognition by macrophages and dendritic cells [52]. Furthermore, PTX-3 interacts with factor H, which activates the alternative pathway of the complement system [53]. High PTX-3 levels during acute NE associate with a severe outcome of the disease, especially severe AKI, thrombocytopenia, and a longer hospitalization [31]. Plasma terminal complement complex SC5b-9 levels correlate with severe thrombocytopenia [15]. Thus, PTX-3 is considered to have a role in the disease pathogenesis because of the cross-linkage of coagulation and complement system activation [6,14,15].

In some German studies, severe thrombocytopenia has been associated with severe PUUV-induced AKI [54,55,56]. However, in a Finnish study with 546 NE patients, no association was found between the severity of thrombocytopenia and AKI [11]. These divergent study results can be explained by rather small study populations in the German studies and different host genetic factors influencing the clinical picture [6,11,23].

## 4. Coagulopathy in PUUV Infection

### 4.1. Endothelial Activation

Endothelial cells in the vasculature of various organs are major targets of hantaviruses. Although hantavirus replication takes place in the vascular endothelium, this does not seem to cause direct cytopathologic effects to the infected endothelial cells in vitro studies [5,57]. Hantavirus antigens are encountered in endothelial cells in HFRS, and in endothelial cells in lung capillaries during HCPS [16,58]. Endothelial cells regulate vascular integrity, as well as hemostasis, thrombosis, inflammation, and angiogenesis. Hantavirus infection alters endothelial responses, resulting in endothelial dysfunction and increased capillary permeability, which is the hallmark of the disease pathogenesis [57]. During the clinical course, this is represented as hemorrhages or oedema, hemoconcentration, and hypotension.

In a Swedish study, levels of endothelial glycocalyx degradation and leukocyte adhesion molecules serving as indicators of endothelial dysfunction and markers of vascular repair were elevated, and correlated with the disease severity of the PUUV infection [22]. Another study suggests an imbalance between factors contributing to angiogenesis and vascular integrity, namely angiopoietin-1 and its antagonist angiopoietin-2 [59]. Thus, the PUUV-induced deregulation of angiopoietin levels may contribute to endothelial dysfunction and disease severity [59].

Regarding the upregulation of proinflammatory cytokines, IL-6, tumor necrosis factor-α (TNF-α), and interferon γ (IFN-γ), all capable of activating the endothelium, have been reported to be elevated in hantavirus infections [35,60]. Proinflammatory cytokines are produced in macrophages or dendritic cells as a response to the recognition of hantaviruses, which cause a change from the anti- to the pro-adhesive phenotype of endothelial cells. Pro-adhesive cells bind monocytes through intercellular adhesion molecule 1 (ICAM-1) and integrin β2–integrin interaction, and platelets through vWF–αIIbβ3 integrin interactions [35]. Elevated levels of soluble endothelial cell receptors, such as E-selectin, ICAM, and tumor necrosis factor receptor (TNFR)-1, are present in acute HFRS [51,61,62]. Finally, activated macrophages and platelets promote coagulation and fibrinolysis and complement and contact pathway activations along with various mediators involved in immune responses [6,35].

The lack of an appropriate animal model has limited the knowledge of hantavirus infection pathogenesis, although macaque monkeys (*Macaca fascicularis*) infected with wild-type PUUV strains are able to produce a disease resembling human PUUV infection [63,64].

#### 4.1.1. Neutrophil Activation

**Figure 1:**
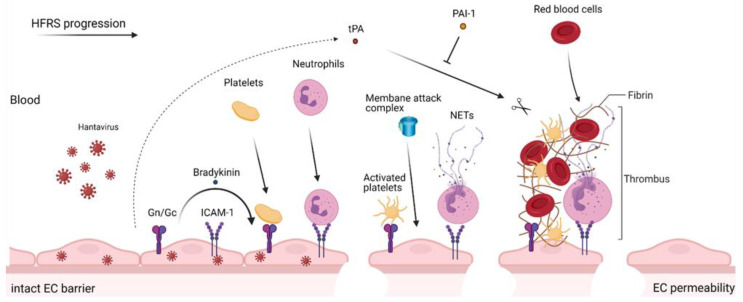
Vasculopathy in HFRS. Hantaviruses infect endothelial cells (EC) lining the vasculature. The infected ECs express viral Gn/Gc glycoproteins and upregulate ICAM-1 adhesion molecules, to which circulating platelets and neutrophils adhere. The interactions activate platelets and neutrophils (through neutrophil extracellular traps, NETs), which promote coagulation through thrombus and fibrin clot formation. At the same time, the infected ECs induce the local production of bradykinin that is released into the bloodstream, which, together with complement membrane attack complex, compromises the EC barrier function, resulting in increased blood flow into tissues. Another factor produced by infected ECs, tissue plasminogen activator (tPA) solubilizes blood clots and thereby contributes to EC permeability. The activity of tPA is inhibited by plasminogen activator inhibitor (PAI)-1 that is upregulated in the more severe HFRS cases (caused by DOBV hantavirus). This image was created with BioRender.com.

Neutrophils, the most abundant blood leukocytes in humans, play an important role in the innate immune response at the sites of infection or inflammation. Neutrophils aim to kill invading microbes by producing reactive oxygen species and releasing antimicrobial proteins, such as myeloperoxidase (MPO) and human neutrophil elastase (HNE), in a process of degranulation [65]. Neutrophils also release neutrophil extracellular traps (NETs), consisting of extracellular chromatin accompanied with histones and granule proteins, that have the potential to entrap and kill pathogens [66]. Increased amounts of circulating histones, cf-DNA, and histone—DNA complexes have been described during acute PUUV infection, suggesting NET formation [47,67,68]. Hantaviruses are capable of inducing NETosis through β2 signaling [67]. We recently found that neutrophil activation is mediated by PUUV-infected endothelial cells [69]. It is known that, when activated, leukocytes bind to endothelial cells via ICAM-1 and transmigrate to tissues. In addition, we reported increased levels of MPO, HNE, histone H3, and IL-8, a chemotactic factor of neutrophils, during acute NE, suggesting neutrophil activation through NETosis and/or degranulation [69]. Neutrophil activation markers were not only associated with the severity of AKI, but also with a low platelet count, leukocytosis, and elevated tissue plasminogen activator (tPA), a marker indicating fibrinolysis [69]. A summary of the vasculopathy in HFRS is presented in Figure 1.

NETosis is also considered to be important in host defense through procoagulant mechanisms. NETs support histones and neutrophil DNA fragments in order to induce coagulation activation during sepsis and inflammation [70]. Histones recruit and promote platelet and endothelial activation, while negatively charged DNA provides an activated surface for the assembly of coagulation factors [71]. Neutrophil elastase released from NETs can inhibit the tissue factor pathway inhibitor (TFP1) [72] and thrombomodulin (TM), thus impairing the protein C pathway and enhancing thrombin generation [73].

In an acute PUUV infection, plasma cf-DNA concentrations, considered to be originated from apoptotic cells or NETs, correlate with prothrombin fragments, F1+2, and APTT, which comprise the screening coagulation tests. In addition, associations with complement activation SC5b-9 and C3 are found [14]. This may imply mutual interactions between NETosis and complement activation. More knowledge has emerged that NETs can serve as a platform for complement activation, while activated complement proteins can also stimulate NET formation [74]. In addition, increased plasma cf-DNA levels associate with PTX3 during acute NE are probably explained by the opsonization and clearance of apoptotic and necrotic cells [47].

#### 4.1.2. Vascular Endothelial Growth Factor, Bradykinin, and Nitric Oxide

Vascular endothelial growth factor (VEGF) induces angiogenesis in the endothelium and may also increases vascular permeability via β3 integrin signaling [75]. VEGF levels are elevated in hantavirus infection, during which, the degradation of vascular endothelial (VE)-cadherin may enhance vascular permeability [76,77,78]. VE-cadherin is an endothelial adhesion molecule that maintains cell contact integrity and regulates vascular permeability via the VEGF receptor 2 (VEGF-R2) [79]. Increased hantavirus-induced permeability has been inhibited by antibodies against VEGF-R2 [80]. However, some studies indicate a role for VEGF in endothelial remodeling and vascular repair rather than dysfunction or damage [22,35,81].

As mentioned above, the vascular leakage of endothelial cells (increased capillary permeability) is another key element in the pathobiology of hantavirus disease. Bradykinin (BK) is an inflammatory peptide that promotes vasodilation, vascular permeability, edema formation, and hypotension. The potent vasodilator BK is generated locally by endothelial cells from HMW kininogen by the kallikrein–kinin proteolytic system [82]. When hantavirus-infected endothelial cells are incubated with plasma proteins involved in the kallikrein–kinin pathway, the kallikrein–kinin system is activated via factor XII and BK production is increased, resulting in an enhanced vascular permeability in vitro [83]. The kallikrein–kinin system activation, with the enhanced synthesis and release of BK, probably contributes to the vascular leakage in vivo. There are two reported cases of severe PUUV-infected patients who were successfully treated with the BK type 2 receptor antagonist, icatibant (a decapeptide similar in structure to BK but containing five nonproteinogenic amino acids), which further supports the role of BK in the disease pathogenesis [68,84,85].

An enhanced nitric oxide (NO) formation associates with elevated TNF-α and with the degree of hypotension in acute NE [50,86]. NO is constitutively formed in the endothelial cells by endothelial NO synthase (eNOS) and in macrophages and neutrophils by inducible nitric oxide synthase (iNOS), mainly in response to inflammatory stimuli and cytokines. NO is important both in the inhibition of platelet activation and the aggregation and promotion of vasodilatation that increases blood flow in the vessels [87]. NO has also been reported to exhibit antiviral effects on the hantavirus replication cycle in cell culture studies and in mouse models in vivo [88]. Endothelial cells can also restrict immune responses through NO release. In addition, cytotoxic T cells may contribute to the capillary damage via immunopathology, caused by the increased release of NO and TNF-α in PUUV infection [50,86].

Host genetic factors influence the clinical outcome of PUUV infection [5,6]. The eNOS gene polymorphism G894T (Glu298Asp) has been reported to associate with clinically severe NE [21]. This eNOS polymorphism (G894T) results in decreased enzymatic activity and decreased basal NO synthesis and release in blood vessels [89,90]. In the kidney, NO produced by mesangial and tubular cells is a significant regulator and protector of renal blood flow, glomerular filtration rate, and tubular function [91]. Those subjects who were TT-homozygous for the eNOS G894T gene variant were more susceptible to severe AKI, hemoconcentration, a higher blood leukocyte count and a longer hospital stay when compared with the other genotypes [21]. This gene variant may play some role in the endothelial and kidney dysfunction during NE, possibly via reduced constitutive NO formation. However, the eNOS G894T variant was not associated with the depth of thrombocytopenia. Furthermore, the G2087A gene polymorphism of inducible NOS has been found to associate with hypotension, especially among the A-allele carriers, during acute NE [21].

### 4.2. Coagulation Activation

Tissue factor (TF), a protein present in subendothelial tissue and circulating leukocytes and platelets, is the major activator of the blood coagulation cascade leading to thrombin formation. TF expression is sustained by proinflammatory cytokines, chemokines, and procoagulative microparticles (MPs), which are phospholipid membrane vesicles shed from various cell types. MPs derived from platelets and erythrocytes can activate thrombin generation in a factor XII-dependent manner [92]. Thrombin plays a crucial role in hemostasis.

A study with PUUV-infected human umbilical vein endothelial cells showed increased TF expression in the endothelium [93]. Coagulation can be induced by both the TF (i.e., extrinsic pathway) and contact system, also known as the plasma kallikrein–kinin system (i.e., intrinsic) pathways. Incubation of plasma proteins with hantavirus-infected endothelial cells has been shown to result in the cleavage of high molecular weight kininogen, an elevation in enzymatic activities of FXIIa, and an enhanced BK liberation, thus indicating plasma kallikrein–kinin system activation [83]. The activation of the contact system can be both procoagulative via the activation of intrinsic coagulation pathway and proinflammatory via the kallikrein–kinin system activation, leading to enhanced BK-mediated responses [94].

PUUV infection causes the activation of coagulation pathways in the acute stage [12,13,95,96]. Increased circulating prothrombin fragments 1+2 (F1+2), the fibrin degradation product D-dimer, and diminished amounts of physiologic anticoagulants, antithrombin (AT), protein C (PC), and protein S (PS), indicate enhanced in vivo thrombin formation [12,14,96]. Thrombin generation may also take place in the shed microparticles in vivo, although no difference in the procoagulant activity of MPs in peripheral circulating blood could be detected when comparing the acute and recovery stages of PUUV infection [41,97]. In a recent study, increased circulating extracellular vesicle (i.e., microparticle) TF activity was observed during NE, which was significantly associated with plasma levels of tPA and PAI-1 [98]. Particularly, the extracellular TF activity was shown to peak in patients with DIC compared with patients who did not have DIC. This suggests that endothelial cells may be the possible source of the procoagulative TF that drives coagulation activation [98].

The thrombin generation assay, calibrated automated thrombogram (CAT) method, can be used to assess the overall coagulation capacity in plasma. The hemostatic balance and thrombin generation in platelet-poor plasma has been evaluated using the CAT assay in acute NE [99]. The in vitro thrombin generation capacity was decreased and the time to peak was prolonged in the acute phase when compared with the recovery phase. Thrombocytopenia and an increased fibrinogen level correlated with the decreased endogenous thrombin potential (ETP) and peak thrombin concentration, which suggests thrombin activation and platelet consumption. Diminished ETP, however, did not correlate with the bleeding symptoms that were reported in one-third of the PUUV-infected patients [99]. Together with the low platelet count, enhanced fibrinolysis, and signs of increased thrombin generation in vivo, this set of data suggests a mild to moderate consumption coagulopathy during acute NE. Thrombocytopenia, loss of physiological anticoagulants, enhanced fibrinolysis, and diminished plasma thrombin generation potential in vitro, shift the overall hemostatic balance toward a hypocoagulable state and bleeding tendency.

### 4.3. Fibrinolysis

Fibrinolysis has been suggested to be enhanced during acute NE. In one study a 24-fold increase in acute phase D-dimer levels was noted [12]. The coagulation variables, F1+2 and D-dimer, also associate with thrombocytopenia and the maximum creatinine level [41]. In addition, tPA is strongly and acutely upregulated, especially in patients with hemorrhages and also in PUUV-infected cynomolgus macaques, as well as in PUUV-infected cultured endothelial cells. In contrast, the urokinase plasminogen activator (uPA) and plasminogen activator inhibitor-1 (PAI-1) level remained unaltered [93,100,101]. PAI-1 functions as the main inhibitor of plasminogen activators and hence fibrinolysis (i.e., physiological breakdown of blood clots). The finding of increased tPA activity most likely reflects the activation of fibrinolysis. Furthermore, interferons can induce tPA expression directly through the signal transducer and activator of transcription 1 (STAT1). Elevated tPA levels are associated with the length of hospital stay, weight gain, minimum platelet count, leukocytosis, and high levels of terminal complement complex, IL-6, and maximum hematocrit level [101]. A study with PUUV-infected human umbilical vein endothelial cells demonstrated that PUUV was also able to increase PAI-1 levels in the acute phase [93]. Furthermore, an increased tPA/PAI-1 ratio has been suggested to contribute to the hemorrhages during acute NE [100,101]. Genetic polymorphism of PAI-1 has also been shown to associate with the severity of AKI among Finnish NE patients [102].

### 4.4. Disseminated Intravascular Coagulation (DIC)

Signs of DIC are common during acute hantavirus infection [12,16,96,103]. However, the applied diagnostic criteria for DIC have varied in different studies. The laboratory abnormalities in this form of consumption coagulopathy include a prolonged activated thromboplastin time (APTT) and PT/international normalized ratio (INR), decreased levels of platelet and fibrinogen, and an increased amount of the fibrin degradation product, D-dimer. The excessive activation of coagulation includes thrombin generation, possibly leading to microvascular thrombosis when overcoming the anticoagulant system. The consumption of coagulation factors and platelets may lead to bleeding seen in DIC.

According to the modified scoring system of the International Society of Thrombosis and Haemostasis (ISTH), DIC was diagnosed in 28% of patients during acute NE [96]. DIC was also associated with a more severe disease. A similar finding was reported in a Finnish study, where DIC was diagnosed in five of the nineteen hospital-treated patients according to the ISTH criteria [12]. However, no associations were found between the positive DIC score and elevated levels of D-dimer and F1+2. In addition, the score was not predictive of the clinical outcome of NE [12]. In Swedish case series studies, acute PUUV infection has been associated with a transient increased risk of acute myocardial infarction and stroke, as well as with venous thromboembolism [8,9].

## 5. Complement Activation

The complement system is a tightly regulated network of proteins bridging innate and adaptive immunity. It plays an important role in host defense, inflammation and in the clearance of microbes and damaged cells and immunocomplexes. It can be activated by three major pathways: the classical, the alternative, and the lectin-dependent. They all converge at complement component C3, which is the main component of the complement in the blood, resulting in the formation of the activation products, C3a, C3b, and C5a and the end product of the complement cascade, the membrane attack complex (MAC) C5b-9. MAC causes the osmotic lysis of the target cells [104]. The complement system is linked to the coagulation and fibrinolytic system via multiple interactions through a complex serine protease system [105].

The complement system is activated in acute NE, which is implicated by increased SC5b-9 levels and decreased C3 [15,106]. Complement activation of the classical pathway associates with a more severe clinical disease, although activation through the alternative route has been shown to be more common [106]. Anaphylatoxins C3a and C5a can contribute to the endothelial cell activation and permeability along with directing MAC-mediated vascular injury [107]. Hantavirus infection induces galectin-3 binding protein (Gal-3BP) production, a glycoprotein reported in chronic viral infections that associates with MAC levels. Excessive Gal-3BP formation is suggested to sensitize the infected cells for complement attack [35]. Complement attack against glomerular endothelial cells may also contribute to kidney dysfunction in hantavirus infection, as Gal-3BP has been found to be produced in the glomeruli and tubular epithelium of PUUV-infected macaques [35].

SC5b-9 can increase endothelial permeability via ligating β3-integrin, releasing BK and platelet-activating factor [108,109]. Additionally, the MAC complex can mediate cellular reactions and the formation of inflammatory cytokines that are able to alter endothelial function. These observations further link complement activation to the impairment of endothelial integrity and vascular permeability.

### Interactions between Coagulation, Predictive Biomarkers and the Complement System

Multiple connections between the host’s inflammatory response and coagulation pathways in NE have been demonstrated. PTX-3, an acute phase reactant produced at the site of inflammation, has been reported to associate strongly with different variables reflecting thrombin formation and the activation of both coagulation, complement, and endothelium in acute NE [14]. Elevated PTX3 levels associate with F1+2 and lowered endothelial marker ADAMTS13. Furthermore, PTX3 associates with the consumption of the platelet ligand, fibrinogen. In addition, PTX3 levels correlate with diminished levels of natural anticoagulants, such as AT, PS free antigen, and PC [14]. PTX3 is known to activate the classical complement pathway by binding the complement component C1q [110], and to interact with P-selectin [111]. It can also interact with factor H, the alternative complement pathway regulator [53]. These are all possible mechanisms linking together PTX3, coagulation activation, and thrombocytopenia. In NE, PTX3 correlates directly with the terminal increased complement component complex SC5b-9 and inversely with the C3 levels [31]. To conclude, these findings emphasize the role of PTX3 in the crosstalk between coagulation, complement activation, and inflammation in the pathogenesis of hantavirus infection.

Antithrombin (AT) functions as a serine protease inhibitor that inhibits coagulation enzymes, especially thrombin and activated coagulation factor X. Furthermore, AT inhibits both classical and lectin pathway complement enzymes. AT also has anti-inflammatory properties [112]. AT induces the endothelial release of prostacyclin and thus suppresses platelet activation and aggregation, inhibits the adhesion of neutrophils to the endothelium, and decreases the production of the cytokines IL-6 and TNF in the endothelial cells. In addition, AT inhibits leukocyte activation by downregulating P-selectin activity [112]. Lowered AT activity, commonly seen in sepsis, can be reduced due to impaired AT synthesis in the liver, AT degradation by proteases, consumption by excessive thrombin formation, and increased vascular permeability [112,113]. Reduced AT activity has been demonstrated to associate with high PTX3, cf-DNA, IL-6 and low plasma C3 during acute NE. These biomarkers have been shown to predict severe PUUV infection characterized by thrombocytopenia, high leukocyte count and longer hospitalization [15,30,31,47]. In addition, low AT activity correlates with low platelet count [12]. The finding of depleted AT activity and biomarkers predicting disease severity is in line with studies reporting correlations between reduced AT activity and poor outcomes in sepsis and critically ill patients [114].

Elevated plasma cf-DNA levels, considered to be originated from apoptotic cells or NETs, associate with thrombin formation (i.e., prothrombin fragments F1+2), reduced AT activity, and complement activation. The capacity of hantaviruses to induce NET formation through β2 signaling has been demonstrated to contribute to the disease immunopathology [67]. In addition, cf-DNA associates with PTX3 in NE, probably explained by the opsonization and clearance of apoptotic and necrotic cells [31]. NETosis is considered to be important in host defense through the innate immune response, but also through procoagulant mechanisms. NETs support histones and neutrophil DNA fragments in inducing coagulation activation during sepsis and inflammation [70,74]. Histones recruit and activate platelets, while negatively charged DNA provides an activated surface for coagulation factors to assemble. Furthermore, neutrophil elastase released from NETs can inhibit the TF pathway inhibitor [72] and thrombomodulin, thus impairing the PC pathway [73]. In addition, reduced complement C3 levels, indicating the alternative pathway activation of the complement system, associate with the loss of natural anticoagulants, PC and PS free antigen, as well as with APTT. This refers to an ongoing interaction between the activated complement and coagulation systems [14]. The main findings regarding the activation of coagulation, endothelial cells, and the complement system are summarized in Table 1.

## 6. Conclusions

Current knowledge refers to multiple interactions between activated coagulation and complement pathways during acute NE. NETosis also plays a role in this triangular relationship. As reviewed above, these findings that are associated with the pathogenesis of PUUV infection are suggestive of immunothrombosis, with the potential to aid innate immunity and host defense against pathogen invasion.

## Figures and Tables

**Table 1:** The main alterations in the laboratory markers of coagulation, endothelial cell activation, and complement system in acute Puumala hantavirus infection.

**Coagulation Markers**	**Acute-Phase**	**References**
APTT	Prolonged	[12]
Prothrombin time	Shortened	[12]
Thrombin time	Shortened	[12]
Fibrinogen	Increased	[12,13]
F1+2	Increased	[12]
D-dimer	Increased	[12]
Antithrombin activity	Decreased	[12]
Protein C activity	Decreased	[12]
Protein S free antigen	Decreased	[12]
Plasma PTX-3	Increased	[31]
Plasma cf-DNA	Increased	[47]
tPA activity	Increased	[69,93,100]
PAI-1	Not altered	[100,101]
**Platelet Ligands and Markers of Endothelial Cell Activation**	**Acute Phase**	**References**
vWF:Ag	Increased	[13]
vWF:RCo	Increased	[13]
Fibronectin	Decreased	[13]
ADAMTS13 activity	Decreased	[13]
**Complement Activation**	**Acute Phase**	**References**
SC5b-9	Increased	[15,106]
C3	Decreased	[15,106]

Abbreviations: APTT = activated partial thromboplastin time, F1+2 = prothrombin fragments, vWF:Ag = von Willebrand factor antigen, vWF:Rco = von Willebrand factor ristocetin cofactor activity, ADAMTS13 = a disintegrin and metalloproteinase with a thrombospondin type 1 domain 13, SC5b-9 = complement protein SC5b-9, C3 = complement protein C3, cf-DNA = cell free DNA, PTX-3 = pentraxin-3, tPA = tissue plasminogen activator, PAI-1 = plasminogen activator inhibitor 1.
